# Inter-organisational collaboration enabling care delivery in a specialist cancer surgery provider network: A qualitative study

**DOI:** 10.1177/13558196211053954

**Published:** 2022-02-07

**Authors:** Cecilia Vindrola-Padros, Angus IG Ramsay, Georgia Black, Ravi Barod, John Hines, Muntzer Mughal, David Shackley, Naomi J Fulop

**Affiliations:** 1 Senior Research Fellow, Department of Targeted Intervention, University College London, London, UK; 2 Senior Research Fellow, Department of Applied Health Research, University College London, London, UK; 3 Principal Research Fellow, Department of Applied Health Research, 4919 University College London, London, UK; 4 Consultant Urological Surgeon, Specialist Centre for Kidney Cancer, 4965 Royal Free London NHS Foundation Trust, London, UK; 5 Consultant Urological Surgeon and London Cancer Urology Pathway Board Director, Department of Urology, 8964 University College London Hospitals NHS Foundation Trust, London, UK; 6 Consultant General & Upper Gastrointestinal Surgeon, 8964 University College London Hospitals NHS Foundation Trust, London, UK; 7 Medical Director, Greater Manchester Cancer and Manchester Academic Health Science Centre, Christie NHS Foundation Trust, Manchester, UK; 8 Professor of Health Care Organisation and Management, Department of Applied Health Research, University College London, London, UK

**Keywords:** inter-organisational collaboration, cancer surgery provider networks

## Abstract

**Objective:**

To explore the processes, challenges and strategies used to govern and maintain inter-organisational collaboration between professionals in a provider network in London, United Kingdom, which implemented major system change focused on the centralisation of specialist cancer surgery.

**Methods:**

We used a qualitative design involving interviews with stakeholders (*n* = 117), non-participant observations (*n* = 163) and documentary analysis (*n* = 100). We drew on an existing model of collaboration in healthcare organisations and expanded this framework by applying it to the analysis of collaboration in the context of major system change.

**Results:**

Network provider organisations established shared goals, maintained central figures who could create and sustain collaboration, and promoted distributed forms of leadership. Still, organisations continued to encounter barriers or challenges in relation to developing opportunities for mutual acquaintanceship across all professional groups; the active sharing of knowledge, expertise and good practice across the network; the fostering of trust; and creation of information exchange infrastructures fit for collaborative purposes.

**Conclusion:**

Collaborative relationships changed over time, becoming stronger post-implementation in some areas, but continued to be negotiated where resistance to the centralisation remained. Future research should explore the sustainability of these relationships and further unpack how hierarchies and power relationships shape inter-organisational collaboration.

## Introduction

Regional reconfigurations of services or ‘major system change’ (MSC) are one approach for healthcare improvement that may be implemented to reduce costs, address workforce issues, centralise expertise, improve clinical outcomes or both.^1–4^ Major system change such as centralisation of specialist care require collaboration by multiple organisations, often in the form of networks, to plan and implement the change and deliver care after implementation.^5,6^

Networks are often seen as decentred with limited top-down leadership, multiple forms of regional authority and as a way to reduce internal competition,^6^ and inter-organisational arrangements between healthcare providers as the solution to fragmented and increasingly sub-specialised care delivery systems.^7–9^ Networks are defined as ‘whole’, beyond dyadic cooperation between individual organisations,^10^ and a network perspective allows us to understand the processes required for the development of network structures, their management and the relationships between these structures and certain outcomes.^11–14^ Previous research has also highlighted the ways in which different leadership models can shape the effectiveness of networks^13^ and contribute to the implementation of MSC.^6^

Inter-organisational collaboration has been identified as one of the key mechanisms enabling care delivery across provider networks.^15–17^ Defined as collective processes created and maintained by various organisations based on a common goal,^15^ inter-organisational collaboration relies on complex intra and inter-organisational interactions to ensure patients flow across multiple healthcare organisations through a series of handovers between professionals, professional groups and healthcare settings.^18^ Interactions might however be complicated by workforce shortfalls, service priority differences, infrastructure mismatch, differences in standards of care, shifting roles in sites and a history of competition or bad relationships across organisations.^19^

In this paper, we explored the challenges of implementing networks to enable care delivery through a naturalistic analysis of inter-organisational collaboration^11,16,17^ in a provider network that had centralised cancer surgery in London, United Kingdom.

### Implementing major system change: Centralising specialist cancer surgery

The provider network under study comprised 12 provider organisations overseeing the provision of cancer services in the wider area London area (the changes in this area were implemented by an organisation called ‘London Cancer’^3,6^), covering a population of 3.2 million. It sought to improve cancer survival rates and patients’ experience of care, increase patients’ access to a wider range of treatment options and participation in clinical trials. One of the main changes introduced by the network was the centralisation of specialist surgical services. This was based on evidence that increased patient volumes in specialist centres would allow greater specialisation of staff, greater experience and expertise across teams working in those centres,^6^ as well as offering more equitable access to a full range of surgical technologies and innovative techniques, while local centres continue to deliver other types of treatment, diagnostic services and follow-up care.

The network used a central leadership team and network managers (performing hybrid clinical/managerial roles), with provider organisations acting as ‘system leaders’ to implement the changes. A Chief Medical Officer (CMO) role was created to oversee the design, planning and implementation of the changes. A newly formed network Board (an independent, skills-based board formed of experts external to the network and chaired by a former cancer patient) was tasked with clinically led recommendations for the model of care; it also oversaw a bidding process for provider organisations applying to become specialist centres, with recommendations agreed by the chief executives and medical directors of the network provider organisations. Some actors across the network, including clinicians and patients, questioned the rationale for the changes, clinical evidence and ways in which the changes were made, including the selection of organisations that would take on the role of system leaders.^6^ Our earlier analysis of the network highlighted the considerable amount of time required to implement MSC. In this paper, we focus on the processes, challenges and strategies used to govern and maintain inter-organisational collaboration between professionals during the implementation process.

## Methods

### Design and conceptual framework

Our analysis was guided by a conceptual framework informed by D’Amour et al.,^14^ who draw from the structuration model of collaboration to consider the multi-dimensional features of collaboration. The framework is divided into four dimensions: shared goals and visions, internalisation, formalisation and governance; these are operationalised in 10 indicators (Table 1). It has been tested in different settings, including across teams, between organisations and across integrated healthcare networks. To our knowledge, our study is the first time the framework has been applied to the study of a provider network. We sought to address the following research questions: (1) Were inter-organisational relationships developed across the network? (2) What types of inter-organisational relationships developed across the network? and (3) Which factors complicated collaboration and which acted as enablers?

**Table 1.** Indicators of collaboration (based on D’Amour et al. 2008)

Dimension	Indicator	Description
Shared goals and vision	Goals	Identifying and sharing common goals is an essential point of departure for a collaborative undertaking.
	Client-centred orientation vs. other allegiances	There can be an asymmetry of interests among partners or a partial convergence of interests. Collaboration will depend on the extent to which these can be negotiated.
Internalisation of interdependencies	Mutual acquaintanceship	It is necessary to create the social conditions that will foster collaboration, particularly through social interaction.
	Trust	Collaboration is possible when organisations have trust in each other’s competencies and ability to assume responsibilities. Trust reduces uncertainty.
Governance	Centrality	The existence of clear and explicit direction can guide action towards collaboration (i.e. through the use of central authorities who can provide a direction and play a strategic role in implementing collaborative processes).
	Leadership	Leadership can have multiple forms and operate at different levels of the organisation. Collaboration depends on the distribution of power and the ability of all organisations to participate in decision-making.
	Support for innovation	Collaboration can be seen as an innovation in itself, as it often involves new activities or dividing responsibilities differently. Collaboration cannot take place without a complementary learning process.
	Connectivity	Connectivity refers to the fact that organisations have places for discussion and constructing bonds. Connectivity allows for rapid and continuous adjustments in response to problems of coordination.
Formalisation	Formalisation tools	Formalisation is an important means of clarifying the various organisations’ responsibilities. Collaboration is facilitated if the actors involved know what is expected of them and what they can expect from others.
	Information exchange	Information exchange is facilitated by the existence of appropriate information infrastructure. Good mechanisms for information exchange reduce uncertainty and increase trust between organisations.

### Data collection

Data collection took place between September 2015 and April 2019 and focused on 10 sites (including specialist and local centres). It included documentary evidence (approximately 100 documents), which was gathered from online resources and from people involved in the planning and implementation of the changes; non-participant observations (163 hours) of meetings, which we recorded in the form of unstructured field notes; and interviews of stakeholders (*n* = 117) involved in the centralisation of cancer surgery. Interview topic guides covered the different stages of the centralisation (Online supplementary Material 1).

Potential interview participants were identified through a review of documentary evidence and observations of the changes and snowball sampling (Table 2). Participants were contacted via email or telephone and provided with a participant information sheet. Interviews were conducted in person or via telephone only with written informed consent. Interviews lasted approximately 50 minutes and were audio recorded then professionally transcribed. Permission to observe meetings was obtained from the meeting Chair in advance. Participants were given the option to opt out of observations. Meetings were sampled purposively to cover all of the clinical pathways included in the study as well as to capture different levels of governance of the services. All documents analysed were in the public domain or obtained from staff who were in charge of implementing the changes.

**Table 2.** Profile of interview participants

**Participant group**	**Number**
Network managers and other network staff members	9
Local context*	11
Patient representatives	4
Urology Pathway Board** members	5
oesophago-gastric (OG) Pathway Board** members	5
OG staff*** from provider organisations (specialist and local centres)	26
Urology staff*** from provider organisations (specialist and local centres)	57

*Includes commissioners (staff involved in the planning and purchase of NHS and publicly funded social care services), academics, staff members from organisations outside of the network, representatives from patient groups

**Pathway boards were led by clinical pathway directors and include representation from patients, primary care and cancer professionals from across the London area.

***Includes surgeons, nurses, oncologists, allied health professionals, pathologists, managers and radiologists.

### Data analysis

Interview transcripts, observation notes and documentary evidence were analysed using thematic analysis.^22^ We carried out an initial familiarisation stage and identified preliminary codes. We then examined these codes in relation to our framework (Table 1). Codes were grouped in relation to the four dimensions of the framework during a first analysis stage. Codes were then re-examined in relation to the 10 indicators of the framework. The final stage of the analysis was used to explore the 10 indicators in relation to the typology of collaboration.

### Ethical approval

The study received ethical approval in July 2015 from the Proportionate Review Sub-committee of the National Research Ethics Service Committee Yorkshire and the Humber-Leeds (Reference 15/YH/0359).

## Results

We found that London Cancer had varying types of collaboration depending on the organisation, professional group and the indicator explored in our conceptual framework (Table 3). We report our findings according to the four dimensions of the framework.

**Table 3.** Inter-organisational collaboration indicators in London Cancer case study and factors that acted as barriers and enablers to collaboration

**Dimension**	**Indicators**	**Evidence of degree of collaboration and changes through time**
Shared goals and vision	Goals	1. London Cancer articulated goals and objectives. For instance, in the case of urological pathways, the Urology Technical Group was formed before pathway boards. The technical group had representation from all Trusts, undertook options appraisals and designed clinical configurations without specific sites being named or chosen as potential centres. The Urology Technical Group comprised radiologists and oncologists as well as surgeons, nurses, etc. 2. Pathway boards were created to drive the changes and operationalise objectives at the pathway level. 3. Some front-line staff ‘owned’ objectives of improved care delivery and outcomes, while others questioned them. 4. There was loss of trust in process felt by some organisations as not everyone agreed with the goals and the mechanisms through which these would be achieved (i.e. centralisation).
	Client-centred orientation vs. other allegiances	1. A patient-centred focus was established at the outset as the main driver for the centralisation. 2. Some staff members valued other allegiances, often involving loyalty to their employing organisation or a commitment to a service or clinic which contradicted the ‘patient-centred orientation’ set out by the London Cancer network.
Internalisation of interdependencies	Mutual acquaintanceship	There were frequent opportunities for becoming acquainted for some professional groups (i.e. surgeons and nurses), but not for others (e.g. radiologists, oncologists, and allied health professionals).
	Trust	1. Trust was still conditional and in early stages of development in some cases. 2. Some organisations viewed specialist centres as not trusting the capacity of local centres to make good decisions in relation to patient care.
Governance	Centrality	1. Central figures such as London Cancer, pathway boards and system leaders sought consensus at network and organisational levels. 2. While each provider organisation had representatives on the board, not all felt they had the same influence.
	Leadership	1. London Cancer played a central role in the design and implementation of the centralisation. 2. New leaders emerged through the pathway boards and within the different organisations of the networks. 3. Some leadership roles were questioned. For instance, specialist centres were expected to take on the role of ‘system leader’, a role that some non-specialist organisations considered as proof that some organisations would be ‘taking over the system’.
	Support for innovation	Sharing of expertise and good practice was sporadic and fragmented despite being a major component of the original ‘offer’ of these changes.
	Connectivity	There were venues for discussion in some professional groups (in the form of network-level meetings or working groups), but not in others.
Formalisation	Formalisation of management processes	1. Processes such as the development of pathways, guidelines, and structures for joint working were established to reach consensual agreements across the networks. 2. Not all organisations were engaged in the development of pathways and guidelines in the same way. 3. In some cases, these processes for reaching consensual agreement needed to be ratified on various occasions to make sure agreement could be obtained and maintained. 4. These processes were often led by specialist centres.
	Information exchange	1. The network experienced incomplete information exchange infrastructure that did not meet the need of users (these problems were more severe during early implementation stages). 2. Changes in the transfer of information were attempted, but the network continued to experience problems.

### Shared goals and vision

Articulating local goals and a vision was one of the starting points for establishing the inter-organisational collaboration. The independent organisation, London Cancer, characterised their vision as a diffuse ‘paradigm shift’, with a general concern for improving outcomes for patients, a focus on early diagnosis, the support of local improvement initiatives, the establishment of higher patient volumes and the creation of large specialist multidisciplinary teams as a means of making improvements in outcomes for patients. This was formulated in verbal and written communications; for example, in an early planning document, the Chief Medical Officer described the main aim of centralisation as:

The configuration of our specialist cancer services in too many smaller centres makes it impossible for our clinical teams to do their best for patients. This is frustrating for everyone; we need a paradigm shift and are convinced by evidence that consolidating complex and specialist cancer services in a small number of world-class specialist centres where all the experts can work together in high volume teams is the way to achieve it. Such teams will also have the capacity to strengthen expertise and access to innovation at local hospitals. *(London Cancer document, 2013)*

Other organisational drivers were described in more informal ways, such as frustrations with the competitive nature of cancer surgery in London, with ‘*centres that were three or four miles away from each other all competing to be the world class centre and it wasn’t necessarily easy to get people to work together*’ (LON2, London Cancer staff member). The provider organisations who were to become specialist centres tended to communicate a shared goal of improving patient outcomes and delivering patient-centred care: *‘the drivers were very much about patient outcomes, clinical expertise, centralising services for the benefits of patients and the clinicians making best use of a very expensive resource really*’ (LON31, manager, oesophago-gastric pathway, specialist centre). Centres that were losing surgical activity were more sceptical of the centralisation benefit and the assumed mechanism for improvement:

*But just a pure centralisation is* … *even in terms of economies of scale, not going to improve things. Because it’s such a big move. [*…*] What makes sense is quality control, in my view, so clinical governance, quality control.* (LON54, consultant, urology pathway, local centre)

Some interview participants agreed with the centralisation and the creation of specialist centres but did not agree with the processes for selecting these sites. They argued that good patient outcomes were being achieved in sites that had not formally been selected as specialist centres.

So we were easily the highest volume, best audited results, best research in the sector that’s why we were particularly upset when renal cancer was given to the specialist centre who had no history of really renal cancer work at all. (LON 47, urology surgeon, local centre)

In this case, the shared goal was compromised by a loss of trust in the process of building the particular collaborative relationship. In other cases, centralisation was not seen as beneficial to all patients as many would be required to travel longer distances to access care. It was believed that this created financial and logistical difficulties for patients and carers and could lead to treatment non-concordance: *‘I feel for the patients [*…*] people living in east London have to travel here, it’s quite hard even for our patients, which are our own patch of patients to come all the way down*’ (LON24, nurse, oesophago-gastric pathway, specialist centre).

Allegiance often involved loyalty to clinicians’ employing organisation or a commitment to a service or clinic they had developed that would be affected by the changes. These loyalties contributed to barriers in inter-organisational collaboration as they tended to promote personal interests and the loss of focus on the shared goal (in this case, the delivery of patient-centred care).

I’m sorry if I appear to be negative but… you have to appreciate that from my point of view. I came here, I built something up over many years and we had very good results and very good outcomes and I had always been led to believe that if you had good results and good outcomes then you would do well, but unfortunately our outcomes have not been considered and everything that I ever built up has been taken away… and I have nothing anymore... *(LON47, surgeon, local hospital)*

### Internalisation of interdependencies

One underlying assumption in studies on organisational collaboration is that professionals need to know each other and have trust in each other’s competencies to develop collaborative relationships.^10^ In our study, many participants whom we interviewed knew each other and had collaborated in some capacity in the past. Clinical staff attended common events and some structures such as pathway boards for urology (which brought together staff from multiple organisations) were present before the centralisation. Some participants had previously worked in other organisations in the network or were working in joint or shared roles across organisations during the time of the study. This allowed them to become acquainted with people working in various settings.

Mutual acquaintanceship was, therefore, strong among certain professional groups; these were mainly those with established networks before the centralisation. Trust was harder to establish as it involved overcoming doubts about the role each clinician should play, feelings of competition and ‘patient ownership’:

You say to the MDT, which includes the surgical team, ‘this patient is actually quite interested in brachytherapy’, and the response is: ‘I don’t care, I want to see the patient here so I can tell him about surgery’. And you do that consistently whoever says that here is effectively dismissing their professional colleague and almost implying that their professional colleague is not capable of counselling a patient adequately. And I think that was the problem so whatever was being said in the MDT the response was always: ‘send the patient here we will talk to them’. And that became the mantra for the last two or three years, just send them, send them here. And that caused a lot of problems. A lot of people were upset about that because it was perceived as very aggressive. *(LON 26, urology surgeon, specialist centre)*

Questions emerged around who should be in charge of providing patients’ information about all treatment options (not only surgery) and clinicians and managers at local centres felt this responsibility was taken over by staff in specialist centres. They saw the importance of their role diminish and they feared for the sustainability of their service:

We have no prostate specialty lead in the hospital now, we have got one renal consultant who is leading our MDT. We have been in talks with [the specialist centre] to see if they would do any partnership work and just look at joint posts. They have been really reluctant, or they have been helpful in as much as they do send us down a doctor on a Tuesday to perform diagnostics here. But we need long term stability, and we don’t have it anymore, so we have got no doctors. *(LON83, nurse, prostate/bladder pathway, local centre)*

### Governance

We found that the central role played by the London Cancer leadership team fostered the development of collaboration across the organisations that formed part of the network. As noted, specialist centres within the network took on the role of system leader to develop and maintain collaborative relationships and ensure the transfer of information, patients and staff:

So it was meeting with the other Trusts, agreeing roles and responsibilities for the referring Trusts and the specialist Trusts regarding exchange of information about patients, transfer of images, agreed means of communication like generic emails at each Trusts for the information to go, the creation of an action plan after the MDT if there were additional things that needed to be done. *(LON18, renal surgeon, specialist centre)*

As implementation progressed, key members of staff (including clinicians and managers) within organisations became involved in decision-making at network level and ensured the maintenance of collaborative relationships that had been created during the early implementation of the changes. For instance, the service leads, normally doctors, of both specialist and local centres participated in pathway-level meetings, which allowed them to obtain information on the pathway as well as represent the particular needs and interests of their organisation. They then acted as a central point of coordination within their service.

Not all organisations felt they had the same degree of power over decision-making processes, despite the establishment of processes for connectivity such as network-level pathway meetings and specialist multidisciplinary team meetings, which brought multiple organisations together to coordinate care. D’Amour et al.^14^ argue that in collaborative relationships, all partners must be able to express their points of view and participate as equally as possible in decision-making processes. However, our data point to perceived power imbalances within the network and these acted as barriers to collaboration as some organisations felt completely left out of decisions regarding care delivery.

Lack of sharing practice and transferring knowledge across organisations played an important role in the perceptions of exclusion outlined above and these acted as barriers to collaboration. When the centralisation was planned in London, the transfer of knowledge between sites was established as one of the original ‘offers’ of the changes but clinicians and managers (particularly those not in specialist centres) felt that the opportunities for sharing practice and transferring knowledge across sites were very limited.

I think that it’s more important to build a better relationship with our referring urologists, so that we can improve and enable them to follow the patients up better. So rather than them seeing us and saying, “Oh, you’re stealing our work”, and then sending us back all of the follow-up, it’s to try and improve relationships with them so that we’re seen as part of a wider team and it’s not a “them and us” thing. *(LON57, renal surgeon, specialist centre)*

Evidence of connectivity also varied in relation to professional groups. For instance, surgeons and nurses tended to report opportunities for working with other members from their professional group more frequently than other groups such as radiologists, oncologists and allied health professionals:

So, the communication in general, and this is true throughout the NHS, is terrible, we don’t have cancer network meetings which are particularly useful or functional, there is so much opportunity to make people feel more involved. The things that I spoke about like getting all the radiologists and the network in a room to discuss good practice that has happened twice in the last five years that I’ve been working here you know, it’s not something that happens regularly right, and it could be and it should be*. (LON 76, radiologist, prostate/bladder pathway, specialist centre)*

### Formalisation of management processes

Formalisation can be explained as clarifying partner responsibilities through the use of formalised management processes such as agreements, protocols and infrastructure for information exchange, that is, shared patient information systems. In London Cancer, patient pathways were developed and agreed by staff from the relevant organisations across the network and clinical guidelines were also jointly developed to ensure the standardisation of care across the network.

The coordination of patient care was also formalised through the establishment of Specialist Multidisciplinary Team meetings that brought together clinicians from specialist and local centres across the network to discuss patient cases and make decisions around the coordination of care. During the early stages of implementation, the establishment of these meetings encountered what some participants referred to as ‘teething issues’. These included technological problems, such as remote access to meetings and cases of missing patient information, that is, unavailable test results or patient details: ‘*there were a few issues at the beginning, the video link didn’t work for quite a long time [*…*] the actual idea of the MDT worked fine, it was more technical issues*’ (LON33, manager, oesophago-gastric pathway, specialist centre).

As implementation continued, meetings began to flow better in the sense that technological issues were resolved, participants became more accustomed to the meetings and MDT coordinators across sites developed strategies for working together and ensuring all the required patient information was available. Problems still remained in relation to the amount of time people could allocate to the meetings, and according to study participants, this was due to the way in which job plans were developed, a factor that might point to limitations in the design of the centralisation as this issue was not anticipated:

Each of the consultants who present they have problems with their job plans and I don’t think the MDTs had been adequately job planned in the network. It’s been an issue. So they can only dial in for 20 minutes. The thinking behind the MDT is that everybody is dialling into, has dialled in together and everybody listens to everybody else’s cases and what usually happens is that […] consultants can only be there for 20 or 30 minutes and so I tend to let them present and then they can go off. It’s supposed to happen, but I know it won’t happen. And I think that’s just a problem with job planning. *(LON26, surgeon, prostate/bladder pathway, specialist centre)*

Collaborative relationships were formalised through discipline-specific groups, that is, surgeons, nurses and allied health professionals, which discussed aspects of care relevant to their professional practice:

We created two London Cancer CNS (Clinical Nurse Specialist) teams, one for bladder, one for prostate and we meet on a regular basis and one of the things we do talk about is our patient pathways, communication. How to make it better? How we as a CNS team can work together and improve things? Because you can’t always rely, or expect the Admin team to do it, they’ve got an enormous amount of jobs. So, I think it’s quite nice, as from CNS to CNS to be able to refer patients or discuss patients and just makes the pathway tighter and much more personal for the patient. So, these two groups have been set up and they’re flourishing, they’re proving to be quite successful. *(LON 30, CNS, prostate/bladder pathway, specialist centre)*

Another way to formalise collaborative relationships between sites was through the creation of joint clinical roles, where clinicians divided their time across two or more hospital sites (often seeing the same patients through these sites). We identified eight shared clinical roles: four surgeons, one nurse, one radiologist and two oncologists. These roles were also seen as a way to improve working relationships between teams with the person concerned moving across sites could share information about the teams and the best people to contact.

Our observations of service-level meetings pointed to the active role played by individuals known as ‘patient navigators’ in coordinating care for patients across multiple provider organisations. This entailed creating relationships with clinicians in other hospitals, getting to know internal processes for processing patient information and handling referrals in other hospitals, and becoming aware of regional-level support groups and programmes, such as social care, transport or patient support groups (fieldnotes).

The mechanisms for information exchange, such as information technology systems, teleconference facilities or shared patient notes, were not fully developed at the time of the study. This was noticeable from our observations of SMDT meetings, which took place during the early implementation stages when people could not join conference calls (fieldnotes December 2015). We noted several instances where patient cases could not be discussed due to missing information, such as pathology reports (fieldnotes).

## Discussion

Inter-organisational collaboration is an intrinsic component of healthcare delivery, yet its ‘active ingredients’ as they are being established and after they have been embedded remain a relatively understudied area of research.^10^ We applied a conceptual framework that analyses the processes, challenges and strategies used to develop and maintain inter-organisational collaboration between professionals in a provider network where services were centralised.

The creation of collaborative relationships was facilitated by the establishment of shared goals, at least by some organisations, attempts to reach consensus in relation to maintaining patient-centred care, the existence of central figures who could drive the centralisation and the promotion of distributed forms of leadership.^23^ Processes for enabling inter-organisational collaboration such as pathway-level meetings, SMDTs, joint clinical roles and discipline-specific meetings were developed over time, with some early ‘teething issues’ along the way, then consolidated into routine practice. However, some processes were still under development towards the end of our study. For instance, while some professional groups such as CNSs had established clear mechanisms for collaboration at the network level, other groups (e.g. radiologists) felt that they rarely met to discuss guidelines or ways to improve care delivery.

These differences point to the need to visualise and study provider networks as dynamic entities, made up of relationships that are dependent on historical factors, in this case, previous connections between members of the same professional group and existing infrastructure, but also recognising the potential for these relationships to evolve into new types of collaboration. In his analysis of integrated care networks, Mitterlechner^24^ found that the relationships and network governance changed repeatedly through repetitive sequences of collaborative inquiry. These sequences allowed the network to address problems in experimental and innovative ways. We also found that collaborative relationships changed in relation to the negotiation of power relations between organisations, where new powerful actors in the form of specialist centres emerged in the role of ‘system leaders’ to drive the centralisation forward.^25^ Specialist centres set the pace of SMDTs and led the development of the new centralised pathways. Although these new leadership roles did not go uncontested, specialist centres adopted a clear role setting out the types of collaborative relationships that would be required throughout the network and maintaining these through time.

We identified key areas that require further development to ensure active collaboration across the four themes. These involved developing opportunities for mutual acquaintanceship across all professional groups; the active sharing of knowledge, expertise and good practice across the network; the fostering of trust; and creation of information exchange infrastructures fit for collaborative purposes.^26^ At the end of our study, study participants across different hierarchies of the network indicated that active work was underway to address challenges in information exchange and the sharing of expertise, but other areas such as lack of trust, mutual acquaintanceship and connectivity had not been addressed yet.

We found that it was not enough for provider organisations to maintain shared goals, it was also important to address different viewpoints on how these goals should be achieved. While all provider organisations aimed to deliver patient-centred care and improve outcomes, not all agreed that the centralisation model proposed for London Cancer was the best way to achieve these aims. Among those who agreed with the centralisation, not all believed that the sites that were selected to act as specialist centres were the best to deliver specialist cancer surgery. Similarly to the processes described in other studies of centralisation,^2, 3^ we found that it took time for organisations to align themselves to the same vision and agree on the goal and the mechanism for achieving the goal (centralisation), and even after centralisation was implemented, some people continued to question its benefits.

Fotler et al.^27^ proposed the concept of ‘incremental inter-organisational relations’, arguing that organisations tend to establish collaborative relationships that require less commitment and have lower risks first, and then move to riskier and resource-intensive relationships. The organisations observed in our study reached consensus in relation to delivering the care that was best for patients but required additional time and the development of other collaboration mechanisms to engage with centralisation. We would add that the process of incremental inter-organisational collaboration identified by Fotler et al. is also dependent on the constant negotiation of power relations and reinforcement of the status quo.^1,28^ In our study, even after the centralisation was implemented, efforts were made to ratify existing pathways, to make sure all sites aligned to the processes set out to coordinate care across the network and to demonstrate the benefits of the changes for patient outcomes. In a way, this last process focused on outcomes was used to demonstrate that embarking on the centralisation was ‘the right thing to do’.

Hierarchies played an important role within the network. Some provider organisations, mainly specialist centres, were seen as more powerful than others as they were able to influence decision-making processes in relation to care delivery. Networks have been traditionally portrayed as devoid of hierarchies, privileging horizontal forms of governance, over vertical ones.^8^ However, the management literature has also highlighted the prevalence of power imbalances in ‘collaborative governance’.^29,30^ We believe that these imbalances and how they are perceived, enacted and experienced in practice, need to be the focus of future research.

A horizontal lens that looks at relationships across professional groups allowed us to identify different degrees of collaboration, identifying some professional groups where collaboration was active and others where it was still under development. One reason for this might be that some professional groups had historically maintained relationships across the network that could be repurposed after the centralisation. This horizontal focus also allowed us to document the movement of staff across sites, and we found that joint clinical roles facilitated inter-organisational collaboration. Yet, towards the end of the study, some of these roles had started to disappear as staff found it difficult to manage the displacement across different geographical locations, dealing with different patient information systems and addressing situations of role strain, that is, the emergence of tensions due to competing responsibilities between organisations. Future research should look at the sustainability of these types of roles.

Our findings have implications for the future planning and implementation of MSC. Inter-organisational collaboration within networks is shaped by a history of interactions between organisations. When planning MSC, early engagement processes, bringing together all relevant stakeholders from across the network, will be important to develop a shared understanding of the goals and ensure collaborative relationships can be established and sustained. Following a model of incremental inter-organisational collaboration, it might be useful to identify aspects of the development of collaborative relationships that can be easily implemented and focus on those initially, for example, administrative processes that are easy to implement or the use of pre-existing groups where collaborations had already been established. More complex aspects of these relationships can then be worked on gradually, based on this ‘track-record’ of collaborative working relationships.

### Limitations

Our study had several limitations. The retrospective nature of some of the interviews meant they could have been influenced by recall bias as a significant amount of the data analysed for the paper was collected shortly after the implementation of the centralisation and processes of inter-organisational collaboration could have been nascent. To reduce the risk of bias, we used documentary evidence to complement interview participants’ narration of past events and observations during meetings. We made an effort to include the views of a large group of stakeholders and maintain an inclusive sampling strategy that was informed by experts, observations and snowball sampling techniques, but we might have missed relevant individuals. Our study analysed the development of inter-organisational collaboration in a specific healthcare area and in an urban setting; additional work is required to explore collaboration in other specialties and contexts. Our analysis dew on the conceptual framework developed by D’Amour et al.,^14^ which has been tested in several healthcare settings. However, other conceptual frameworks might shed light on aspects of collaboration that we did not explore, such as the shaping of collaboration by pre-existing hierarchies and power relations.

## Conclusions

We have explored the processes, challenges and strategies used to create and maintain inter-organisational collaboration between professionals in a provider network that centralised cancer services. The provider organisations in the network we studied reached consensus in relation to shared goals, maintained central figures who could create and sustain collaboration and promoted distributed forms of leadership. These were dynamic processes still in transformation in the period under study. Organisations still encountered barriers or challenges in relation to developing opportunities for mutual acquaintanceship across all professional groups; the active sharing of knowledge, expertise and good practice across the network; the fostering of trust; and creation of information exchange infrastructures fit for collaborative purposes.

We observed the changes collaborative relationships underwent over time, becoming stronger post-implementation in some areas as consensus was reached over different aspects of care delivery, but continuing to being negotiated in others where resistance to centralisation remained. We examined the variation of inter-organisational collaborative relationships between professional groups and the processes implemented by staff who had joint roles across multiple organisations. Future research should explore the sustainability of these collaborative relationships and identify the factors that might prompt changes in approaches to collaboration used in networks of provider organisations.

## Supplemental Material

sj-pdf-1-hsr-10.1177_13558196211053954 – Supplemental Material for Inter-organisational collaboration enabling care delivery in a specialist cancer surgery provider network: A qualitative study Click here for additional data file. Supplemental Material, sj-pdf-1-hsr-10.1177_13558196211053954 for Inter-organisational collaboration enabling care delivery in a specialist cancer surgery provider network: A qualitative study by Cecilia Vindrola-Padros, Angus Ramsay, Georgia Black, Ravi Barod, John Hines, Muntzer Mughal, David Shackley and Naomi Fulop in Journal of Health Services Research & Policy
