# Supplementary material for: Inter-organisational collaboration enabling care delivery in a specialist cancer surgery provider network: A qualitative study
Source: J Health Serv Res Policy. 2022 Feb 7;27(3):211–21. doi: 10.1177/13558196211053954 (PMC9277336; doi:10.1177/13558196211053954)
Supplement: sj-pdf-1-hsr-10.1177_13558196211053954 – Supplemental Material for Inter-organisational collaboration enabling care delivery in a specialist cancer surgery provider network: A qualitative study [file sj-pdf-1-hsr-10.1177_13558196211053954.pdf]

*Journal of Health Services Research & Policy*
**Inter-organisational collaboration enabling care delivery in a specialist cancer surgery provider network: a qualitative study**
Vindrola-Padros C, Ramsay A, Black G, Barod R, Hines J, Mughal M, Shackley D, Fulop N

## Online Supplement 1

### *Interview topic guides*

### Background

- Can you tell me a bit about your current role and how long you have worked here?
- Where have you worked previously, and in what settings? How long have you been involved in cancer care?
- Can you describe the services provided here for people with cancer?

### Overview of the reorganisation of specialist cancer surgery services

- How did you hear about proposals to reorganise specialist cancer surgery services across London/Greater Manchester? What did you think of these proposals? What did your colleagues think?
- What were the drivers for change (national policy/evidence, local people/organisations)?
- Were you consulted about the proposals to reorganise? Who else was consulted? Were there others who should have been consulted?
- Were there any particular obstacles or barriers to change?
- Were there any particular ‘enablers’ of change?
- Were you kept informed about the proposals as they progressed? How were you kept informed?

### Planning and implementing the changes in your service

- What were services like here before the reorganisation took place?
- Can you describe the changes that took place as a result of the reorganisation?

### *Planning*

- Were you involved in the planning of the changes here? How (preparation of bid, planning of implementation, other activities, timing of these activities)?
- Who else participated in the planning of the services? Who led the planning and how was it governed?  Were other people kept involved or consulted?
- Were staff generally kept up-to-date about the changes as they were being planned?
- Were there any particular obstacles to planning (e.g. staff concerns about the changes in patient pathways)?
- Were there any particular enablers to planning (e.g. examples of good practice in keeping people informed/on board)?

### *Implementation*

- I would like to ask you about the changes that happened here with reorganisation, how they were implemented, and how you were involved. So can you tell me about any of the following?
    - **Processes** (e.g. services and therapies, protocols/standard operating procedures, operation of MDTs): can you describe the changes that happened here, and what had to be done to support them? Did your Trust lose any services as a result of the

reconfiguration? What impact did this loss of services have on Trust activity? Staff retention? Other services?
  - **Staffing** (numbers/rota/skill mix): what was done in terms of staffing to support these changes?
  - **New roles** (or more specialised roles): were any new roles created when reorganising the service, or have new roles developed over time?
  - **Skills and training**: what kinds of training have you or your colleagues received or might you need to support your work in the new services?
  - **Becoming part of a wider system**: how have things changed in terms of how your service interacts with other parts of the local health system? Other parts of the hospital/other hospitals/units/primary care. Would you say that your Trust operates within a network of providers? How is this network managed? How do providers collaborate across the network?
  - **Governance:** have any groups been set up here to oversee and support the changes you've mentioned, or to support high quality care more generally (within the service, within the Trust, across the whole system)?
  - **Financial implications:** how were changes financed? Any unanticipated costs or resource requirements? Any additional funding needed?

***General follow ups to the above***

- What was its purpose? Why was it important?
- What was the background to this? Whose idea was it, and who was involved in agreeing it? What factors influenced these changes/decisions?
- How was this developed? How did it work (e.g. how it was led, who was consulted, how was it planned)?
- How were you involved? How did people work together to develop and implement these changes?
- What factors made a difference when implementing it? Were there any problems? How were these addressed?

**Overall impact of changes to specialist cancer surgery services**

- Overall, in what ways do you think the reorganisation has made a difference to cancer surgery services here? For example, can you tell me about any of the following?
  - **Throughput:** patient volumes, theatre capacity, inpatient bed capacity, imaging, laboratories, etc.
  - **Outcomes** (mortality and morbidity).
  - **Patient and carer experience** (including quality of care and patient choice).
  - **Care provision** (ways of working for staff, training and professional development, deskilling, skilling up).
  - **How service interacts with other hospital departments/other hospitals** (trauma, inpatient wards, anesthetics, imaging, beds).

***General follow ups to the above***

- How is this measured?
- Why and by whom?
- Who is it reported to?
- How is the information used?

**Reflections**

- What lessons have you learned from this? Is there anything you would have done differently?
- Do you think improvements could have happened without reconfiguring services?

- What advice would you give to other services undergoing similar reconfigurations?
- Any further comments/anything else you wish to add?

**Governance-level interviews**

*Note: This is a summary of general topics: some will be of limited or no relevance to certain of our interviewees.*

- What is your current role/post and what does it involve?
- How are/were you involved with the reconfiguration of cancer surgery services?
    - How are/were you involved with the reconfiguration of individual pathways?

**Can you tell me about the proposals to reconfigure specialist cancer surgery?**

- How did you first hear about these plans? What did you think? And your colleagues?
- What were the catalysts or drivers for the reconfigurations? How was the decision made? Why did people decide to change?
    - *national policy? local drivers? key players?*
- Who was consulted on these changes?
- What were the catalysts or drivers for the pathways? Why did people decide to change?
- Were there any prior attempts to try to reconfigure specialist cancer surgery?

**Can you tell me how the reconfigurations were organised?**

- Which groups and individuals led and governed the reconfigurations?
    - *Prompt: who else participated?*
- What were the roles and responsibilities of these groups and individuals?
    - *Prompt: London Cancer, commissioners, London Cancer Alliance, patient groups, pathway leads, Integrated Cancer System*
- What were the resources allocated for the reconfigurations?
    - *Prompt: funding for London Cancer, other types of funding, staff*
    - *Was this funding enough? Too much?*
    - *Did additional funding need to be obtained?*
- How were you involved?
- What were some of the key meetings and events?
    - How did these meetings and events work?
    - What were some of the challenges encountered at these meetings and events?
- What was the overall timeline for the reconfigurations?
    - *Were there any factors that drove the timelines?*
    - *In case of pathway leads, prompt for specific timeline of pathways as some pathways moved at a different pace*

**Can you tell me about how the new models of care were developed and agreed?**

| **Components of change** | **General prompts** |
|---|---|
| Case for change<br>Model of care<br>Service specifications and patient pathways<br>Specialist/local site recommendations | How were you involved? Time dedicated? Time dedicated by support staff?<br>What were the key influences?<br>Obstacles/enablers<br>What did you/your colleagues think?<br>What were some of the challenges? |

**Can you tell me about how the changes are being/will be implemented?**

  - *Prompts: in refocusing services, changes in activity, building capacity, changes in referral pathways, ensuring new governance processes, managing demand and capacity*
  - *Prompts: for Urology, for OG.*
- How are/will you be involved in implementing the changes?
- Which groups and individuals are/will be central to implementation? How do they/will they work?
- How are/will local stakeholders be kept up to date on progress of the reconfigurations?
- How will the process be overseen?
- Obstacles and enablers-how are/will these be addressed? What are/will be the levers for change?
- Are there/will there be any implementation costs or resource requirements that were not anticipated during the planning stages of the reconfigurations?

**What changes will be brought about by the reconfigurations?**

  - *Prompts: organisation, service delivery, partnership working, patient outcomes, costs, patient and carer experience (choice and continuity of care, problems such as issues with travel), staff experience (emphasise ways of working, skill mix and approaches to collaboration)*
- What are the outcomes you expect from the reconfigurations? Why do you think these will be produced? How will they be produced?
- How will they be measured? What capacity is/will be dedicated to collecting these data? Are these measures reliable?
- Do you think these changes will be sustained?
- Would these changes have happened anyway?
- Have the resources put into the design and implementation of the reconfigurations been worthwhile in the short term? Long term?
- Are there any negative outcomes/impacts? Or unanticipated outcomes/impacts?

**How has the delivery of cancer care in the London Cancer area changed as a result of the reconfigurations?**

**Have you been able to identify the impact or changes in outcomes as a result of the reconfigurations?**

**Are you considering any changes in this post-implementation phase?**

**What are the challenges currently faced by providers delivering cancer care in London?**

**How are you addressing some of these challenges? Prompt:**

- Meeting waiting targets
- Staffing shortages
- Adherence to pathways
- Local follow-up

- Diagnosis delays, etc.

**What lessons have you drawn from this? Is there anything you would have done differently?**

- What are the factors that have acted as barriers/enablers?
- What are the challenges that still remain?
- Do you think improvements could have happened without reconfiguring services?
- What advice would you give to other services undergoing similar reconfigurations?
- Any further comments/anything else you wish to add?
